# Robotic versus Mini-Laparoscopic Colposacropexy to Treat Pelvic Organ Prolapse: A Retrospective Observational Cohort Study and a Medicolegal Perspective

**DOI:** 10.3390/jcm13164802

**Published:** 2024-08-15

**Authors:** Valentina Billone, Giuseppe Gullo, Girolamo Perino, Erika Catania, Gaspare Cucinella, Silvia Ganduscio, Alessandra Vassiliadis, Simona Zaami

**Affiliations:** 1 Obstetrics and Gynaecology Unit, AOOR Villa Sofia Cervello Hospital, University of Palermo, 90133 Palermo, Italy; valentinabillone@gmail.com (V.B.); girolamoperino@gmail.com (G.P.); erika.catania0702@gmail.com (E.C.); gaspare.cucinella@unipa.it (G.C.); gandusciosilvia@gmail.com (S.G.); 2 Department of Health Promotion, Mother and Child Care, Internal Medicine and Medical Specialties (PROMISE), University of Palermo, 90133 Palermo, Italy; alessandra.vassiliadis@unipa.it; 3 Department of Anatomical, Histological, Forensic and Orthopedic Sciences, “Sapienza” University of Rome, 90133 Rome, Italy; simona.zaami@uniroma1.it

**Keywords:** POP (pelvic organ prolapse), colposacropexy (CS), mini-laparoscopy, robotic surgery, evidence-based guidelines

## Abstract

**Background**: POP (pelvic organ prolapse) involves the descent of one or more pelvic organs downwards with or without protrusion from the vaginal opening, caused by the relaxation and weakening of ligaments, connective tissue, and pelvic muscles. Such an outcome negatively impacts the quality of life. The gold standard procedure for repairing apical compartment prolapse is colposacropexy (CS) to secure the anterior and posterior walls of the vagina to the anterior longitudinal sacral ligament, located anteriorly to the sacral promontory, using a mesh. Several surgical approaches are feasible. Laparotomic or minimally invasive methods, including laparoscopic or robotic ones, can restore the horizontal axis of the vagina and typically involve concomitant hysterectomy. **Methods**: This study is based on 80 patients who underwent CS at Palermo’s Ospedali Riuniti Villa Sofia-Cervello from 2019 to 2023. Women aged 35–85 at the time of surgery were divided into two groups: 40 patients underwent mini-laparoscopic surgery, and 40 patients underwent robotic surgery. The following parameters were accounted for: demographic data (initials of name and surname, age), preoperative clinical diagnosis, date of surgery, surgical procedure performed, estimated intraoperative blood loss, duration of surgical intervention, length of hospital stay, postoperative pain assessed at 24 h using the VAS scale, and any complications occurring in the postoperative period. Mini-laparoscopic CS (Minilap) and robotic CS (Rob) were then compared in terms of outcomes. **Results**: In the Minilap group, 11 patients out of 40 had a preoperative diagnosis of vaginal vault prolapse. The average age in this group was 61.6. Five of these patients had isolated cystocele, while the rest presented vaginal stump prolapse linked to cystocele, rectocele, or both. The remaining 29 patients in the Minilap group had a preoperative diagnosis of uterovaginal prolapse, also associated with cystocele, rectocele, or both, or isolated in nine cases. In the Rob group (average age: 60.1), 13 patients were diagnosed with vaginal prolapse (isolated or associated with cystocele), while the remaining 27 had a diagnosis of uterovaginal prolapse. In the Minilap group, the average procedure duration was 123.3 min, shorter than the Rob group (160.1 min). **Conclusions:** The data collected throughout this prospective study point to the mini-laparoscopic approach as being preferable over the robotic one in terms of surgical procedure length, intraoperative blood loss, postoperative pain, and aesthetic outcome. Hospital stay duration and post operative complication rates were similar for both groups. The innovative and ever-progressing nature of such procedures calls for novel standards prioritizing patient care as well as medicolegal viability.

## 1. Introduction

POP (pelvic organ prolapse) involves the descent of one or more pelvic organs downwards with or without protrusion from the vaginal opening, caused by the relaxation and weakening of ligaments, connective tissue, and pelvic muscles. Women affected by prolapse experience symptoms that significantly disrupt quality of life and severely limit daily activities [1]. A POP classification was articulated in 1994 by De Lancey, according to the biomechanical features defining the degree or level at which such injuries take place [2]. Level I comprises injuries to uterosacral ligaments, enterocele, and prolapse of the uterus, cervix, or vaginal vault (apical defect); level II encompasses involvement of the vesicovaginal or rectovaginal fascia, cystocele, and rectocele, and such anterior injuries can be further differentiated based on injury localization, whether in the vesicovaginal fascia into central, which can lead to the formation of smooth cystocele without vaginal rugae, or lateral placement, typically exhibiting visible rugae within the cystocele [3]. Such a distinction is highly relevant in terms of outlining a specific reconstructive therapeutic pathway. Lastly, level III defects affect ureterocele, urethral-supporting ligaments, and the perineum [4].

Surgical treatment of POP is the most common gynecological procedure in women over 70 years old. An increasingly high prevalence is foreseeable given growing life expectancies [5]. Such demographic dynamics point to the importance of optimizing surgical approaches to manage this condition effectively. The gold standard procedure for repairing apical compartment prolapse is colposacropexy [6] (CS), which involves securing the anterior and posterior walls of the vagina to the anterior longitudinal sacral ligament, located anteriorly to the sacral promontory, using a mesh. This procedure restores the horizontal axis of the vagina and typically involves concomitant hysterectomy. This surgical procedure can be performed using various approaches: laparotomic or minimally invasive, including laparoscopic or robotic methods. Today, traditional laparoscopy and robot-assisted surgery have grown considerably popular due to their reduced invasiveness and shorter postoperative hospital stays [7]. Different studies have reported fewer hospitalization days, shorter recovery times, and less postoperative pain, with short-term effectiveness comparable to the laparotomic approach [8,9,10,11]. Some retrospective studies have also found a significant reduction in blood loss (60 to 150 mL less) [7,9]. Laparoscopic and robotic CS, in addition to the general advantages of minimally invasive techniques, also offer more precise identification of retroperitoneal structures (presacral vessels, ureters, iliac veins), greatly reducing the risk of intraoperative complications. Proficiency in laparoscopic techniques, both in dissection and suturing, as well as precise knowledge of anatomical structures, not only determines the successful outcome of the procedure but also its duration. Given the growing prevalence of POP among older women and the associated benefits of minimally invasive techniques, laparoscopic and robotic approaches constitute an excellent choice for managing this condition. Not only can such methods address the anatomical issues effectively, but also enhance patient comfort and recovery, which makes them well-suited to the needs of an aging population.

The aim of our retrospective study is to compare the laparoscopic approach (particularly mini-laparoscopic) with the robotic approach for colposacropexy. The aspects we evaluate include intraoperative blood loss, duration of the procedure, length of hospital stay, and postoperative pain. We have also evaluated postoperative complications and esthetic outcomes at one, four, and six months.

## 2. Materials and Methods

We collected data from 80 patients who underwent CS at the Department of Gynecology of the Ospedali Riuniti Villa Sofia-Cervello in Palermo, Italy. The study period covered five years, from 2019 to 2023. Women aged between 35 and 85 years at the time of surgery were included and divided into two equivalent groups: 40 patients underwent mini-laparoscopic surgery, and 40 patients underwent robotic surgery. The assignment to the surgical approach group was random, since all patients exhibited comparable clinical and instrumental characteristics. We created a database in which these patients were recorded, evaluating the following parameters: demographic data (initials of name and surname, age), preoperative clinical diagnosis, date of surgery, surgical procedure performed, estimated intraoperative blood loss, duration of surgical intervention, length of hospital stay, postoperative pain assessed at 24 h using the VAS scale, any complications occurring in the postoperative period (defined according to the Clavien-Dindo classification as any condition requiring intravenous therapy, blood transfusions, or surgical, radiological, or endoscopic procedures), and esthetic outcome. We then extracted the results from this database to evaluate any differences between the Minilap group (CS with mini-laparoscopic approach) and the Rob group (CS with robotic approach). Our analyses aimed to demonstrate the superiority of the mini-laparoscopic approach over the robotic approach in terms of surgery duration and esthetic outcome, and their similarity in terms of postoperative pain. All patients included in the database underwent a thorough medical and obstetric history and preoperative gynecological examination: a transvaginal ultrasound was performed to exclude uterine and adnexal pathologies, a routine Pap smear, and, importantly, a vaginal examination to stage the extent of uterovaginal prolapse, using the POP-Q System, and to assess the extent of cystocele and any associated rectocele. This clinical examination was performed both at rest and under effort, with the patient performing the Valsalva maneuver. Additionally, patients with concomitant bladder dysfunction or urinary incontinence underwent preoperative urodynamic evaluation. The patients enrolled in the study did not exhibit significant signs and symptoms related to posterior compartment dysfunction that would require a specialized proctological evaluation. The main preoperative symptoms recorded were mixed urinary incontinence, recurrent cystitis, and genital discomfort. The diagnostic inclusion criterion was symptomatic POP of stage II–IV according to the POP-Q classification and, in particular, the presence of uterine or apical prolapse treatable with colposacropexy. Additional inclusion criteria regarding patients’ clinical and demographic characteristics were: (1) body mass index (BMI) up to 38; (2) ASA (American Society of Anesthesiology) class lower or equal to III; (3) absence of pregnancy or acute pelvic inflammatory disease (PID); and (4) absence of liver disorders or coagulation disorders. The exclusion criteria were: (1) presence of advanced stage malignancy assessed by imaging exams; (2) uterine size exceeding the 16th week of gestation (approximately 1390 g) on pelvic ultrasound examination; (3) previous midline laparotomy; (4) clinical history or ultrasound evidence of severe endometriosis; and (5) presence of severe cardiopulmonary comorbidities contraindicating the performance of pneumoperitoneum and Trendelenburg position. All patients were informed about the type of surgery they would undergo, with the signing of informed consent. Preoperative antibiotic prophylaxis was performed before surgical intervention. General anesthesia was administered to all patients. Postoperative analgesia was provided by administering an opioid (morphine or tramadol) and ketorolac via elastomeric pump. Administration of 1 g of paracetamol and/or one vial of ketorolac was performed only if requested. All patients received thromboembolic prophylaxis with subcutaneous enoxaparin 12 h after surgical intervention, continued in the following days according to the patient’s thrombotic risk. Patients were discharged only when they achieved full mobility, adequate urinary and bowel function, and absence of fever.

For the conventional mini-laparoscopic approach, 3 mm instruments (Karl Storz^®^, Tuttlingen, Germany) such as scissors, forceps, monopolar electrocoagulators, bipolar scissors for tissue dissection and coagulation, and an aspiration and irrigation system were used. In cases of concomitant hysterectomy, a uterine manipulator (Clermont-Ferrand, Karl Storz^®^) was used. The Da Vinci Si^®^ system from Intuitive Surgical was used for robotic procedures. Both techniques were performed with the patient in the lithotomy position, catheterized, and with the arms secured along the body. Pneumoperitoneum induction at 15 mmHg was performed with a Veress needle. In the robotic approach, the optical port (12 mm) was inserted at the level of the umbilicus, and under direct visualization, the other trocars were inserted: two 8 mm trocars for instruments placed 10 cm laterally and 30° downward from the optical port, respectively, on the right and left. The second operator’s trocar (5 mm) was positioned approximately 8 cm laterally to the right accessory trocar and a few cm inferior to the equator of the optical port. In the mini-laparoscopic technique, the central trocar was placed at the umbilical level, and three ancillary trocars of 3 mm were placed in the right iliac fossa, left iliac fossa, and suprapubic region, always under visualization. After trocar placement, a Trendelenburg position of at least 20° was set to induce the ascent of the viscera by gravity and thus free the operative field.

Promontosacropexy, when deemed necessary, involves hysterectomy as the first surgical step, with possible bilateral adnexectomy based on age and comorbidities. Anterior dissection is then performed up to the bladder trigone (the patient is catheterized before the start of the procedure), and posterior dissection is performed up to the rectovaginal space. At this point, a polypropylene mesh with dimensions of approximately 3–4 cm in width and 5–10 cm in length is inserted. It is shaped intraoperatively by the surgeon based on sacro-vaginal distance, extent of prolapse, and dimensions of the uterus/cupola. Once inside the abdominal cavity, it is secured to the vaginal vault by two or three sutures. The promontory of the sacrum is then identified. Using an electrosurgical knife, the peritoneum overlying the promontory is longitudinally incised; the presence of vascular structures such as the bifurcation of the vena cava and the right iliac vein, as well as the right ureter, makes this step extremely delicate. Particular attention is also paid to the middle sacral vessels that originate directly from the aorta and vena cava and descend along the promontory of the sacrum. Ensuring hemostasis, the incision is continued inferiorly below the promontory for 5–6 cm, staying laterally to the sigmoid and medially to the right ureter; the sacral periosteum is then exposed, and the free end of the mesh is secured to the anterior longitudinal ligament, located anteriorly to the promontory of the sacrum, with two or three non-absorbable sutures. Sutures placed too inferiorly (S3 or S4) are more at risk of presacral hemorrhage, while those placed too high could excessively deviate the vaginal axis or cause discitis. Once the mesh is anchored, the peritoneum is closed using absorbable sutures (a step called ‘peritonization’).

After completing the promontosacropexy, depending on the patient’s clinical picture, a Burch colposuspension is performed. With the Retzius space open, access to the bladder neck and periurethral tissues is possible. Two absorbable sutures are placed through the endopelvic and vaginal fascial complex with the help of the second operator, who supports the vaginal fornices with a finger. These sutures are then anchored to the Cooper ligament at a distance of approximately 2–4 cm from the vagina.

## 3. Results

In the Minilap group, out of 40 patients, 11 had a preoperative diagnosis of vaginal vault prolapse. The average age of the group was 61.6. Five of these patients had isolated cystocele, while the remaining presented vaginal stump prolapse associated with cystocele, rectocele, or both. The remaining 29 patients in the Minilap group had a preoperative diagnosis of uterovaginal prolapse, also associated with cystocele, rectocele, or both, or isolated in nine cases. Moving to the Rob group (with an average age of 60.1), 13 patients had a preoperative diagnosis of vaginal prolapse (isolated or associated with cystocele), while the remaining 27 had a diagnosis of uterovaginal prolapse.

We began our analysis by evaluating the duration of surgical intervention in minutes: in the Minilap group, the average duration was 123.3 min, significantly shorter compared to the 160.1 min of the Rob group. It is worth noting that shorter surgical durations lead to reduced time under anesthesia, which can decrease the risk of anesthesia-related complications and contribute to a quicker overall recovery for the patient. As for estimated intraoperative blood loss in milliliters, in the Minilap group, half of the patients completed the intervention with EBL ≤ 50 mL (reported as ‘traces’ in our records). Additionally, in the Rob group, 20 patients had intraoperative EBL ≤ 50 mL. Therefore, after accounting for the remaining patients in the Minilap group, the average intraoperative blood loss in mL was 130 mL. This average was 113 mL in in the Rob group.

The smaller incisions used in mini-laparoscopic surgery and the precision of laparoscopic tools often lead to less trauma to surrounding tissues, which can contribute to reduced intraoperative blood loss. On the other hand, robotic systems offer high-definition visualization and enhanced dexterity, which can theoretically reduce bleeding. However, the learning curve and longer setup times can offset these advantages, especially in the hands of less experienced surgeons.

The visual analogue scale (VAS) to assess postoperative pain was administered 24 h post-surgery, with values ranging from 0 (absent pain) to 10 (the strongest pain imaginable). In the Minilap group, the average postoperative pain score reported by patients was 3.55. In the Rob group, however, the average VAS score was 4.82.

The length of hospital stay for patients operated on with a mini-laparoscopic approach was on average 4.4 days, the same as for those operated on with robotic surgery.

Reviewing postoperative complications, we recorded only a few cases classified as grade I according to the Clavien-Dindo classification: in the Minilap group, two patients developed fever, resolved with the administration of antipyretics alone. One patient complained of abdominal pain, and another presented with mild postoperative anemia (preoperative hemoglobin 11 g/dL vs. postoperative 7.9 g/dL), treated with iron therapy.

In patients who developed postoperative anemia, blood transfusions were not administered, since their hemoglobin levels at admission were within the normal range (12–16 g/dL). Upon discharge, iron therapy and a follow-up complete blood count (CBC) were prescribed. In the Rob group, two patients developed fever, one of whom required blood cultures, and an episode of deep vein thrombosis was diagnosed by Doppler ultrasound of the lower limbs, for which therapy with sodium fondaparinux was initiated.

At the one month post-intervention follow-up, no particular complications were observed; the aesthetic appearance of the surgical wounds was also evaluated. The 3 mm trocars allowed for rapid healing, free from complications with excellent aesthetic outcomes confirmed also at the three and six month follow-ups in the Minilap group. In the group that underwent robotic CS, surgical wounds at 30 days were more evident due to the larger diameter trocars (8 mm).

While robotic surgery offers advanced technological benefits and precision, these advantages may not sufficiently outweigh the benefits provided by the laparoscopic approach, especially as far as surgical duration, intraoperative bleeding, postoperative discomfort, and cosmetic results are concerned. Therefore, based on these outcomes, the laparoscopic approach (particularly mini-laparoscopic) appears to be the more favorable option.

## 4. Discussion

The first open CS was performed in 1950 [12] and was considered the gold standard treatment for genital prolapse [13]; however, the first laparoscopic colposacropexy was described in 1992 [14]. Robotic colposacropexy began to gain ground as a viable procedure from 2004, and several authors immediately extolled its short learning curve, reduced intraoperative blood loss, greater ease in suturing, and shorter associated hospital stay [9,15,16,17,18]. Similar to our prospective study, several scientific publications preceding ours have reviewed the surgical technique of colposacropexy. Several studies have already drawn a comparison between the laparoscopic and the robotic approaches. Seror et al. [19], for example, published a prospective study to compare short-term functional outcomes obtained after laparoscopic and robot-assisted laparoscopic sacrocolpopexy. The authors detected a statistically significant difference in the duration of bladder catheterization, which was shorter in the robotic group, with a median of 2 days, compared to a median of 3 days in the laparoscopic group. They also found no significant disparity between the two groups based on the Clavien-Dindo classification grading, and no discrepancies were observed regarding analgesia usage or length of hospital stay between the two groups [18]. Another paper that compared conventional laparoscopic and robotic-assisted laparoscopic sacrocolpopexy for vaginal apex prolapse was published by Paraiso et al. [20]. This single-center blinded randomized trial involved 78 participants with stage 2–4 posthysterectomy vaginal prolapse. Participants were randomly assigned to laparoscopic or robotic sacrocolpopexy. The primary outcome taken into account was the total operative time from incision to closure. Secondary outcomes include postoperative pain, functional activity, bowel and bladder symptoms, quality of life, anatomic vaginal support, and healthcare system perspective costs [19]. The total operative time was significantly longer in the robotic group compared to the laparoscopic group (a difference of 67 min; *p* < 0.001). Anesthesia time, total time in the operating room, total sacrocolpopexy time, and total suturing time were all significantly longer in the robotic group. Participants in the robotic group also reported significantly higher pain levels and required a longer duration of nonsteroidal anti-inflammatory drug usage. Additionally, the robotic group incurred higher costs than the laparoscopic group. However, both groups demonstrated significant improvement in vaginal support and functional outcomes one year after surgery, with no notable differences between the groups [19].

Joubert et al. [21] also compared laparoscopic and robotic approaches for CS, but in a narrower patient category: those with obesity (BMI ≥ 30 kg/m^2^). Theirs was a comparative retrospective multicenter study on 39 obese patients treated with a laparoscopic approach, and 17 treated with a robotic one. The authors evaluated length of operation, associated procedures, complication rate, and length of hospitalization. They found no significant difference between the groups regarding the requirement for concomitant procedures, such as subtotal hysterectomy or mid-urethral sling placement. The operative time and perioperative complication rate was comparable across both groups [21].

Another aspect that has been examined in the currently available research data concerns the learning curve of the two different surgical approaches. While many surgeons presume that robotic-assisted surgical techniques are easier to learn compared to advanced laparoscopic skills, several studies have suggested that this advantage may apply primarily to novice surgeons [22,23]. Many surgeons have in fact been known to employ robotic-assisted laparoscopy as a transitional stage toward mastering advanced conventional laparoscopy.

In light of the various distinctive features of each patient and complexities which need to be accounted for when choosing a surgical course of action for treating POP, the role of evidence-based guidelines and best practices is of utmost importance. The three possible approaches for sacrocolpopexy (open, laparoscopic, and robotic) should be discussed thoroughly by the doctor in an accurate and exhaustive consultation with the patient. Such a discussion has to take into account that data for the assessment of complications are still inconclusive for all three surgical alternatives, particularly in the long term. Most studies herein accounted for reflect a degree of uncertainty as to the evaluation of effect, which shows that in most cases the sample size was small, the event rate was low, and the study period was relatively short. Additionally, cost implications for concomitant treatment of stress urinary incontinence were not reported [24]. Raising awareness among patients as to the high incidence of pelvic organ prolapse can contribute to countering stigma and psychological and social factors that can prevent timely diagnosis [25]. The need to shed a light on symptoms, expected workup, and treatment options has been stressed by the International Urogynecology Association (IUGA) and the American Urogynecologic Society (AUGS) [26]. Just as importantly, a degree of objectivity and clarity as to POP staging can be provided by the broadly used pelvic organ prolapse quantification system (POP-Q) [27]. Such a tool was devised through a consensus document for staging pelvic organ prolapse at the 1996 International Continence Society, by the American Urogynecologic Society and the Society of Gynecologic Surgeons [28]. Treatment choice in fact largely depends on the extent to which POP affects the patient’s quality of life. Not every POP case needs surgery or any other form of treatment [29]. Throughout the process of evaluating a therapeutic pathway, a thorough and individually tailored assessment, based on a validated urinary incontinence-specific symptom and quality-of-life questionnaire, is essential from a medicolegal standpoint as well. The potential of POP to bring about life-changing, albeit not life-threatening, consequences and damage can in fact lead to diminished capabilities (in a work setting, for instance), in turn resulting in litigation and negligence-based malpractice charges if the decision-making and informed consent phases are not adequately implemented, and negative outcomes ensue. Such actions can involve doctors, facilities, and even manufacturers of pelvic mesh implants in case of design defects or lacking information and warnings about the risks such procedures may entail. Two 2022 Australian class actions [30] were settled by major manufacturers when an Australian court denied arguments that manufacturers should be excused from liability regarding risks or complications that should be known to doctors, or which doctors and surgeons are able to discover, and warn of, themselves. The court found that while medical goods cannot all be risk-free, patients should expect those devices to carry appropriate warnings about risks, even if those risks are rare. This is only one instance of several such cases on the record over the past twelve years [31]. In addition to such crucial points, it is worth highlighting the complexities arising from fast-moving innovations and advances set to revolutionize the way healthcare is delivered over the next years [32]. Specifically, robotic surgery has given rise to the pressing need to redefine the legal, regulatory, and ethical standards we have relied on for a long time [33]. Relevant research has focused on the still inconclusively determined aspects of such approaches, among which three certainly stand out: the lack or inadequacy of uniformly standardized training and practicing for robotic surgery, which is arguably a potential risk to both patient safety and surgical expertise. Just as importantly, a cornerstone of medicolegal viability, the informed consent process, calls for a reconfiguration in order to make absolutely sure that patients are made aware of such innovative technologies in terms of their capabilities, benefits, and risks. The very notion of legal liability itself takes on an extra layer of complexity because of the inherent functional features of robotic systems, which need the involvement of several novel operators and stakeholders, including the manufacturers of increasingly complex systems. As such systems acquire ever higher levels of autonomy, and as the development of artificial intelligence and machine learning forge ahead, new liability and ethical/legal dilemmas loom on the horizon [32,33,34,35,36].

## 5. Conclusions

The data collected through our retrospective study point to the laparoscopic approach (particularly mini-laparoscopic) as the preferable option over the robotic approach, particularly in terms of surgical procedure length, intraoperative blood loss, postoperative pain, and esthetic outcome. The laparoscopic approach has a higher level of efficiency, speed, and technical ease of execution, mostly thanks to the streamlined nature of the laparoscopic instruments and techniques, which require fewer setup steps and adjustments during the procedure. Furthermore, mini-laparoscopic instruments are designed to be more intuitive, thus easier to handle. Surgeons often find that the familiarity and direct manipulation of these instruments facilitate faster surgical execution. On the other hand, robotic surgery involves a more complex setup process, including the docking of the robot, calibration, and adjustment of the robotic arms. Such a degree of complexity can make the procedure lengthier and even entail a longer learning curve for surgeons to master robotic systems, although currently available findings are still inconclusive.

Although average hospital stay length and postoperative complication rates were similar between the two approaches, careful monitoring and management of complications remain crucial. Adherence to evidence-based guidelines and patient assessment based on specifically tailored standards are essential from a clinical as well as a medicolegal perspective. It is imperative that such unprecedented challenges do not catch us unprepared, and the necessary level of readiness is only achievable through a concerted multidisciplinary effort originating from scholars, policy- and law-makers, and advocacy groups and organizations representing the patients. Future research should explore factors influencing postoperative outcomes and the long-term effectiveness of different surgical approaches. Promoting continuous collaboration and adaptability to new evidence and technologies is essential to guarantee the highest quality of patient care as well as a sufficient degree of objectivity, and hence medicolegal viability, of increasingly structured and complex procedures, through novel, updated evidence-based standards.

## Data Availability

All data are available upon request to the corresponding author.
